# Ramelteon Reduces Oxidative Stress by Maintenance of Lipid Homeostasis in Porcine Oocytes

**DOI:** 10.3390/antiox11091640

**Published:** 2022-08-24

**Authors:** Jing-Tao Sun, Jin-Dong Yuan, Qi Zhang, Xin Luo, Xin-Yue Qi, Jia-Hui Liu, Xi-Qing Jiang, Sanghoon Lee, Anukul Taweechaipaisankul, Zhong-Hua Liu, Jun-Xue Jin

**Affiliations:** 1 Key Laboratory of Animal Cellular and Genetics Engineering of Heilongjiang Province, College of Life Science, Northeast Agricultural University, Harbin 150030, China; 2 Laboratory of Theriogenology, College of Veterinary Medicine, Chungnam National University, Daejeon 34134, Korea; 3 National Nanotechnology Center (NANOTEC), National Science and Technology Development Agency (NSTDA), Pathumthani 12120, Thailand

**Keywords:** ramelteon, oocyte maturation, oxidative stress, lipid homeostasis, pig

## Abstract

This study aimed to determine the underlying mechanism of ramelteon on the competence of oocyte and subsequent embryo development in pigs during in vitro maturation (IVM). Our results showed that the cumulus expansion index was significantly lower in the control group compared to the ramelteon groups (*p* < 0.05). Moreover, supplementation of 10^−11^ and 10^−9^ M ramelteon significantly increased the cumulus expansion and development-related genes expression, and reduced apoptosis in cumulus cells (*p* < 0.05). In oocytes, the nuclear maturation rate was significantly improved in 10^−11^, 10^−9^, and 10^−7^ M ramelteon groups compared to the control (*p* < 0.05). Additionally, the level of intracellular GSH was significantly increased and ROS was significantly decreased in ramelteon-supplemented groups, and the gene expression of oocyte development and apoptosis were significantly up- and down-regulated by 10^−11^ and 10^−9^ M ramelteon (*p* < 0.05), respectively. The immunofluorescence results showed that the protein levels of GDF9, BMP15, SOD1, CDK1, and PGC1α were significantly increased by 10^−11^ M ramelteon compared to the control (*p* < 0.05). Although there was no significant difference in cleavage rate, the blastocyst formation rate, total cell numbers, and hatching/-ed rate were significantly improved in 10^−11^ M ramelteon group compared to the control (*p* < 0.05). Furthermore, embryo development, hatching, and mitochondrial biogenesis-related genes were dramatically up-regulated by 10^−11^ M ramelteon (*p* < 0.05). In addition, the activities of lipogenesis and lipolysis in oocytes were dramatically increased by 10^−11^ M ramelteon compared to the control (*p* < 0.05). In conclusion, supplementation of 10^−11^ M ramelteon during IVM improved the oocyte maturation and subsequent embryo development by reducing oxidative stress and maintenance of lipid homeostasis.

## 1. Introduction

It is well known that lipid droplets (LDs) are storage organelles at the center of lipid homeostasis and energy balance [1]. They are evolutionary-conserved organelles found in almost all organisms, from bacteria to mammals [2]. Originating from the endoplasmic reticulum, LDs can associate with most other cellular organelles through membrane contact sites [3]. In oocytes, lipid primarily consists of triglyceride and specific fatty acids which differ by species, stored in distinct droplet organelles [4]. Due to the LDs undergoing considerably spatial, morphological, and dynamic variations during oocyte maturation [5,6], so they play key roles in the regulation of cellular lipid homeostasis. However, generally, in vitro-produced embryos are frequently associated with mitochondrial dysfunction and high reactive oxygen species (ROS) level due to elevated lipid contents compared to in vivo environment [7], which could induce DNA damage and change cell signaling and cellular functions. High fat diet induced metabolic abnormalities, resulting in developmental defects in human and mouse oocytes [8,9]. In bovine, high palmitic acid exposure induced lipid accumulation and decreased the development and quality of oocytes by increasing oxidative stress [10]. Moreover, Aizawa et al. demonstrated that maintenance of LDs is essential for mouse preimplantation embryo development [11]. Therefore, the balance between lipogenesis and lipolysis might interact with the redox signaling during oocyte maturation [12,13]. In addition, there is 161 ± 18 ng of endogenous lipids in a porcine oocyte, which is much higher than other species [14]; therefore, it is an ideal models for studying lipid and fatty acid metabolism in female gametes [12].

Ramelteon has been approved by the Food and Drug Administration (FDA) for the treatment of insomnia. It is a melatonin receptor agonist with a unique mechanism of action that selectively targets specific melatonin receptors MT1 and MT2 [15]. In comparison to the other newly introduced drugs, ramelteon appears to be well-tolerated, and the safety of ramelteon has been examined in numerous preclinical and clinical trials [15,16]. It works by mimicking melatonin, but unlike melatonin which is nonselective for all receptors. Therefore, the specific targeted ramelteon can be considered promising agents for understanding the molecular mechanism of MT1 and MT2 receptor signaling [17]. Nevertheless, limited information is available on the effects of ramelteon on mammalian oocyte maturation during In vitro maturation (IVM). Taken together, we hypothesize that ramelteon targets melatonin receptor to maintain lipid homeostasis and reduce oxidative stress during porcine oocyte maturation. In the current study, we therefore, for the first time, investigate the effects of ramelteon on oocyte maturation, and clarify the underlying regulatory mechanisms during porcine oocyte maturation in vitro. To evaluate the effects of ramelteon on oocyte maturation during IVM, we conducted experiments with five chemical treatments: control (non-treatment), and 10^−11^, 10^−9^, 10^−7^, and 10^−5^ M ramelteon.

## 2. Materials and Methods

### 2.1. Chemicals

All chemicals and reagents were purchased from Sigma-Aldrich Chemical Company (St. Louis, MO, USA), unless otherwise stated.

### 2.2. Porcine Oocyte In Vitro Maturation

Porcine ovaries were placed into a thermos and transported to the laboratory at 30–34 °C from the local slaughterhouse, and the 3–6 mm diameter follicles were aspirated using a syringe with an 18-guage needle. Cumulus-oocyte complexes (COCs) were collected with several cumulus cells layers, and washed 3 times with tissue culture medium-199 (TCM-199; Invitrogen, Carlsbad, CA, USA) containing 10 mM HEPES, 1% penicillin-streptomycin and 0.3% polyvinyl alcohol (PVA); Finally, COCs were placed into IVM medium (TCM-199 containing 0.91 mM sodium pyruvate, 10% porcine follicular fluid, 10 IU/mL follicle stimulating hormone and 10 IU/mL luteinizing hormone). Selected 50 COCs/condition (with 0, 10^−11^, 10^−9^, 10^−7^, and 10^−5^ M ramelteon) was then cultured in an incubator at 5% CO_2_, and 38.5 °C for 42 h.

### 2.3. Assessment of Cumulus Cell Expansion

Briefly, the cumulus expansion index (CEI) was performed as described by Jin et al. [12]. After 42 h IVM, CEI was distinguished into five grades. Grade 0 (no cell expansion): cumulus cells degenerated and formed a flattened monolayer of fibroblast-like morphology; Grade 1 (no cell expansion): porcine oocyte with compacted cumulus cells (consistent with GV stage); Grade 2: the most out layers of cumulus cells exhibited expansion. The CEI grade 3 complexes observed intact expansion, except corona radiata; Grade 4: cumulus cells exhibited intact expansion in all cell layers.

### 2.4. Detection of Nuclear Maturation

After 42 h IVM, cumulus cells were denuded from the porcine COCs using 0.1% hyaluronidase, and oocytes were distinguished into following categories under bright field of microscope: metaphase II (MII, with the 1st polar body extrusion), immature or degenerated.

### 2.5. Assessment of Intracellular GSH and ROS Levels in Oocytes

The level of glutathione (GSH) and ROS in matured oocytes were detected using CellTracker Blue CMF2HC (Invitrogen) and H2DCFDA (Invitrogen). Each sample was exposure in 10 µM CellTracker Blue and 10 µM H2DCFDA for 30 min at room temperature and then placed into 4 µL PBS (285 ± 5 mOsm). Using an epifluorescence microscope (TE2000-S; Nikon, Tokyo, Japan) the stained samples were imaged, and the mean fluorescence intensity of individual oocyte was measured using ImageJ software (version 1.46r; National Institutes of Health, Bethesda, MD, USA). Fluorescence signals were quantified from at least 30 individual oocytes per each group for one biological repetition.

### 2.6. Parthenogenetic Activation (PA) and In Vitro Culture (IVC)

Porcine oocytes with the 1st polar body were suffered from electrical activation (condition: a single direct current pulse of 1.5 kV/cm for 60 µs). Following the activation, the oocytes were incubated with porcine zygote medium 3 (PZM-3) at 38.5 °C at 5% CO_2_ for 7 days. The rate of cleavage and blastocyst was observed on Day 2 and Day 7, respectively. To determine the total cell numbers, the blastocysts on Day 7 were stained with 10 μg/mL Hoechst33342 for 4 min, and the samples mounted on glass slides with a coverslip, and captured with a fluorescence microscope.

### 2.7. LD, Fatty Acid (FA), and ATP Staining

Fluorescence staining was performed as described by Jin et al. [18]. Porcine oocytes with 1st polar body were fixed in 4% paraformaldehyde (PFA) overnight at 4 °C, and then placed into DPBS supplemented with 10 µg/mL BODIPY-LD (BODIPY 493/503; D3922; Molecular Probes, Eugene, OR, USA) for LDs, 6 µM BODIPY-FA (BODIPY 558/568 C12; D3835; Molecular Probes, Eugene, OR, USA) for FAs, or 500 nM BODIPY-ATP (BODIPY FL ATP; A12410; Molecular Probes, Eugene, OR, USA) for 1 h. Following, samples were washed 3 times and mounted on coverslips. Using an epifluorescence microscope (TE2000-S; Nikon, Tokyo, Japan) the stained samples were imaged, and the mean fluorescence intensity of individual oocyte was measured using ImageJ software (version 1.46r; National Institutes of Health, Bethesda, MD, USA). Fluorescence signals were quantified from at least 30 individual oocytes per each group for one biological repetition.

### 2.8. Immunofluorescence Staining

Briefly, immunofluorescence staining was modified as described by Yin et al. [19]. Porcine oocytes were fixed in 4% PFA for 1 h, then washed 3 times and permeabilized with 1% Triton X-100 for 30 min. Following, samples were blocked with 2% bovine serum albumin (BSA)–PBS overnight at 4 °C. Next day, samples were incubated in primary antibody (GDF9, ab93892, Abcam, Cambridge, UK; BMP15, PA5-34401, Invitrogen, CA, USA; SOD1, ab13498, Abcam, Cambridge, UK; CDK1, ab18, Abcam, Cambridge, UK or PGC1α, ab54481, Abcam, Cambridge, UK). Thereafter, a donkey anti-mouse IgG (H + L) highly cross-adsorbed secondary antibody (1:200; Alexa Fluor 546, A10036, Invitrogen, Carlsbad, CA, USA) or a goat anti-rabbit fluorescein isothiocyanate-conjugated secondary antibody (1:200; Jackson Immuno Research Laboratories Inc., West Grove, PA, USA) was applied for 2 h, respectively. Following, samples were washed 3 times and mounted on coverslips. Using an epifluorescence microscope (TE2000-S; Nikon, Tokyo, Japan) the stained samples were imaged, and the mean fluorescence intensity of individual oocyte was measured using ImageJ software (version 1.46r; National Institutes of Health, Bethesda, MD, USA). Fluorescence signals were quantified from at least 30 individual oocytes per each group for one biological repetition. For ease of comparison, the average expression level of each protein from the control group was set as 1.

### 2.9. Real-Time Polymerase Chain Reaction

The cumulus cells or oocytes total mRNA were extracted from 50 COCs for each replicate according to the manufacturer’s protocol. The samples were placed into TRIzol reagent (Invitrogen), and the total mRNA concentration was quantified using a NanoDrop 2000 Spectrophotometer (Thermo Fisher Scientific, Wilmington, DE, USA). Thereafter, production of complementary DNAs (cDNAs) was using amfiRivert cDNA Synthesis Platinum Master Mix (GenDEPOT, Barker, TX, USA). A PCR plate was made by adding 10 µL SYBR Premix Ex Taq (TaKaRa, Otsu, Japan), 8.2 µL Nuclease-free water (NFW; Ambion, Austin, TX, USA), 0.4 µL (10 pmol/µL) forward primer, 0.4 µL (10 pmol/µL) reverse primer, and 1 µL cDNA, and then amplified on a StepOneTM Real-Time PCR System (Applied Biosystems, Waltham, MA, USA). The gene expression was quantified relative to the housekeeping genes *GAPDH* and *RN18S* using R = 2^−ΔΔCt^ equation. The primer sequences are presented in Table S1.

### 2.10. Statistical Analyses

Data are described as mean ± SEM. Significant differences were determined using the Duncan test following a parametric one-way ANOVA with the statistical software SPSS 19.0 (SPSS Inc., Chicago, IL, USA). The gene expression of oocytes and blastocysts, and fluorescence staining of oocyte were compared by Student’s *t*-test. Differences with *p* < 0.05 were considered statistically significance. More than 30 mature oocytes were used to a kind of analysis for per replicate. Each experiment was repeated at least three times.

## 3. Results

### 3.1. Ramelteon Improved Cumulus Expansion

**Figure 1:**
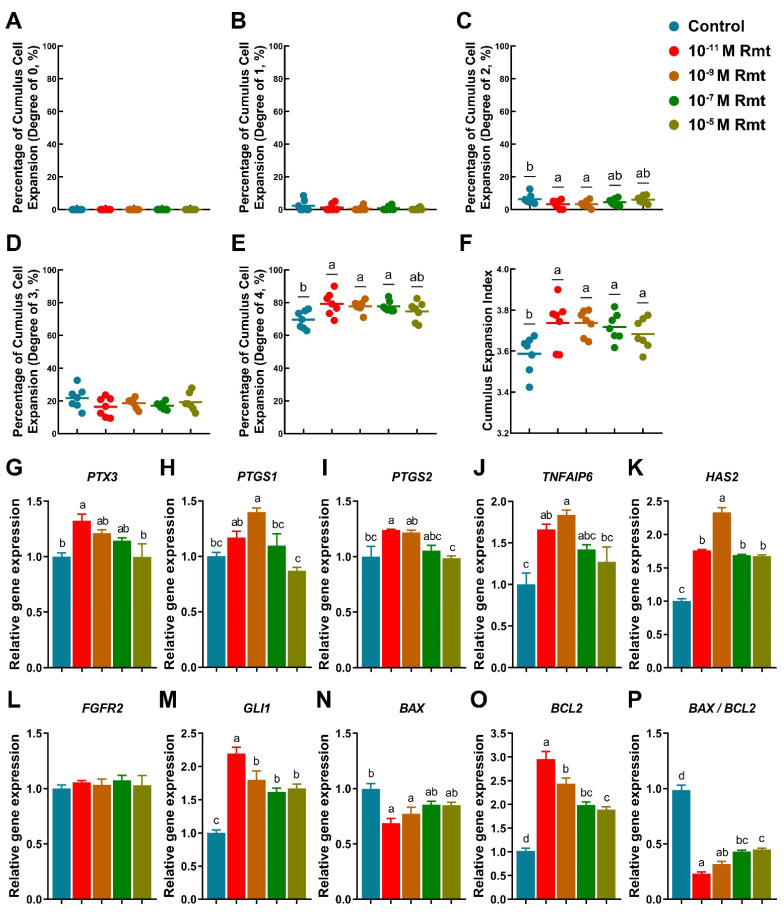
Cumulus expansion index and expansion-related gene expression. (**A**–**F**) Different degree of cumulus cells expansion index; Expression of cumulus expansion- (**G**–**K**); development- (**L**,**M**); apoptosis-related genes (**N**–**P**). Different letters denote significant difference (*p* < 0.05). Results are shown as the average ± SEM of at least three repeats of independent experiments. Rmt, Ramelteon.

Our results showed that treatment of 10^−11^, 10^−9^ and 10^−7^ M ramelteon could significantly improve the proportion of COCs exhibiting complete cumulus expansion compared to the control (CEI grade of 4; 79.29 ± 2.66%, 77.79 ± 1.32%, and 77.64 ± 1.25% vs. 69.56 ± 2.05%, respectively; *p* < 0.05; Figure 1E). Contrarily, 10^−11^ and 10^−9^ M ramelteon groups significantly reduced the proportion of oocyte with CEI grade of 2 compared to the control (3.14 ± 0.95% and 3.12 ± 0.81% vs. 6.35 ± 1.17%, respectively; *p* < 0.05; Figure 1C). Overall, the average CEI was notably increased in all ramelteon groups compared to the control (3.74 ± 0.04, 3.74 ± 0.02, 3.71 ± 0.03 and 3.68 ± 0.03 vs. 3.59 ± 0.03, respectively; *p* < 0.05; Figure 1F).

On the other hand, the expression of cumulus expansion-related genes (*PTX3*, *PTGS2*, *TNFAIP6*, and *HAS2*) were significantly increased in 10^−11^ M ramelteon, and the expression of *PTGS1*, *TNFAIP6* and *HAS2* were significantly increased in 10^−9^ M ramelteon compared to the control (Figure 1G–K; *p* < 0.05). Although the oocyte development-related gene *FGFR2* was no significant difference between groups, *GLI1* was significantly higher in ramelteon groups than control group (Figure 1L,M; *p* < 0.05). Additionally, the expression of *BAX* was significantly decreased and *BCL2* was significantly increased in 10^−11^ and 10^−9^ M ramelteon groups compared to the control (Figure 1N,O; *p* < 0.05), thereby the ratio of *BAX*/*BCL2* was significantly decreased in 10^−11^ and 10^−9^ M ramelteon groups (Figure 1P; *p* < 0.05) in cumulus cells.

### 3.2. Ramelteon Improved Oocyte Maturation

**Figure 2:**
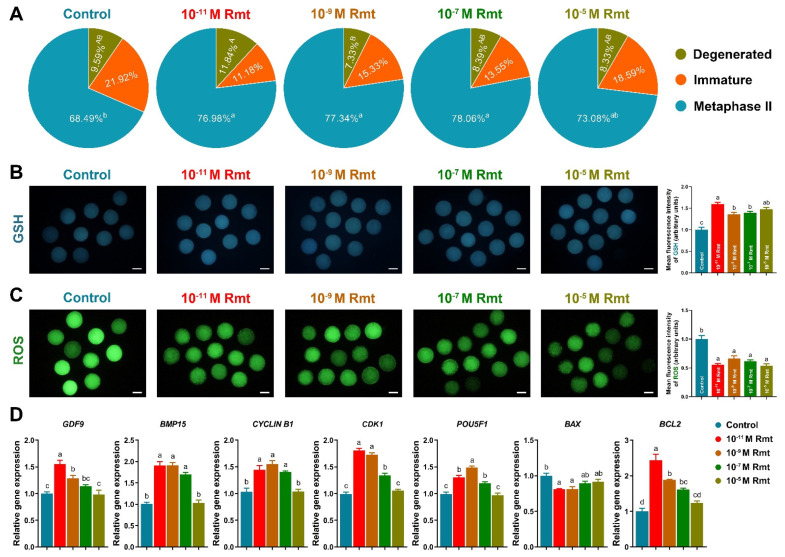
Effects of ramelteon on porcine oocyte nuclear and cytoplasmic maturation. (**A**) Nuclear maturation rate; (**B**,**C**) level of GSH and ROS; (**D**) oocyte development-related gene expression. Different letters denote significant difference (*p* < 0.05). Results are shown as the average ± SEM of at least three repeats of independent experiments. Rmt, Ramelteon. Scale bars = 100 μM.

As Figure 2A showed that treatment of 10^−11^, 10^−9^ and 10^−7^ M ramelteon significantly increased oocyte maturation rate compared to the control (77.2 ± 2.5%, 77.5 ± 3.6% and 78.3 ± 2.7% vs. 68.7 ± 3.3%, respectively; *p* < 0.05). Moreover, all ramelteon groups significantly increased intracellular GSH level and decreased ROS level in oocytes (Figure 2B and C; *p* < 0.05). The oocyte development-related genes (*GDF9*, *BMP15*, *CYCLIN B1*, *CDK1*, *POU5F1*) and *BCL2* were significantly increased, and *BAX* was significantly decreased in 10^−11^ and 10^−9^ M ramelteon groups compared to the control (Figure 2D; *p* < 0.05).

### 3.3. Ramelteon Improved Embryo Development after Parthenogenetic Activation

**Figure 3:**
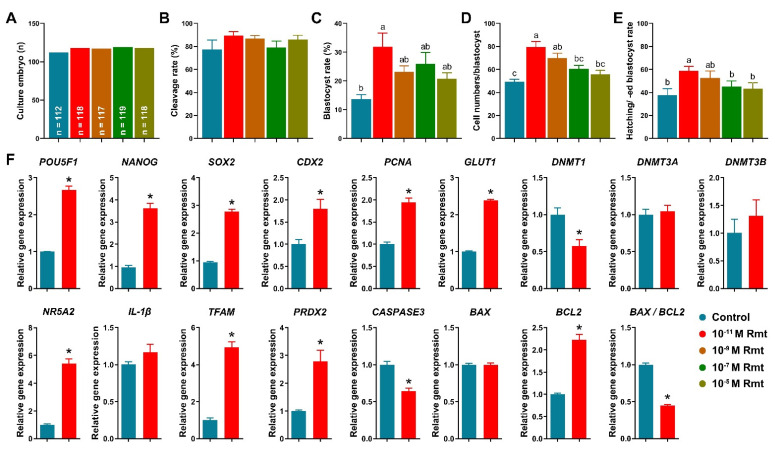
Early embryonic development after parthenogenetic activation and embryo development-related gene expression. (**A**) Culture number of embryos; (**B**) cleavage rate; (**C**) blastocyst formation rate; (**D**) total cell numbers per blastocyst; (**E**) hatching/-ed blastocyst rate; (**F**) Expression of embryo development- and apoptosis-related genes in blastocyst. Within the same indicator, different letters or asterisks denote significant difference (*p* < 0.05). Results are shown as the average ± SEM of at least three repeats of independent experiments. Rmt, Ramelteon.

Figure 3A–E showed that treatment of 10^−11^ M ramelteon significantly improved blastocyst formation rate (31.9 ± 4.7% vs. 13.7 ± 1.6%), total cell numbers of blastocyst (79.6 ± 4.7% vs. 49.3 ± 2.3%), and hatching/-ed blastocyst rate (58.7 ± 3.9% vs. 37.8 ± 5.6%) compared to the control (*p* < 0.05). However, there was no significant different in cleavage rate (89.3 ± 3.3% and 77.5 ± 7.8%). Based on these results, we compared the control and 10^−11^ M ramelteon groups for subsequent experiments.

Our results showed that 10^−11^ M ramelteon group significantly increased the expression of embryo development-related genes, including *POU5F1*, *NANOG*, *SOX2*, *CDX2*, *PCNA* and *GLUT1*, and significantly decreased the expression of *DNMT1* compared to the control (*p* < 0.05). Though there was no significant difference in the expression of *DNMT3A* and *DNMT3B* in blastocyst between groups (Figure 3F). Furthermore, 10^−11^ M ramelteon groups significantly increased the expression of blastocyst hatching- (*NR5A2*) and mitochondrial-related genes (*TFAM* and *PRDX2*; *p* < 0.05). Moreover, the expression of *CASPASE3* and *BAX/BCL2* ratio were significantly decreased, but the expression of *BCL2* was significantly increased in 10^−11^ M ramelteon treated group compared to the control (*p* < 0.05).

### 3.4. Ramelteon Participated Maintenance of Lipid Homeostasis

**Figure 4:**
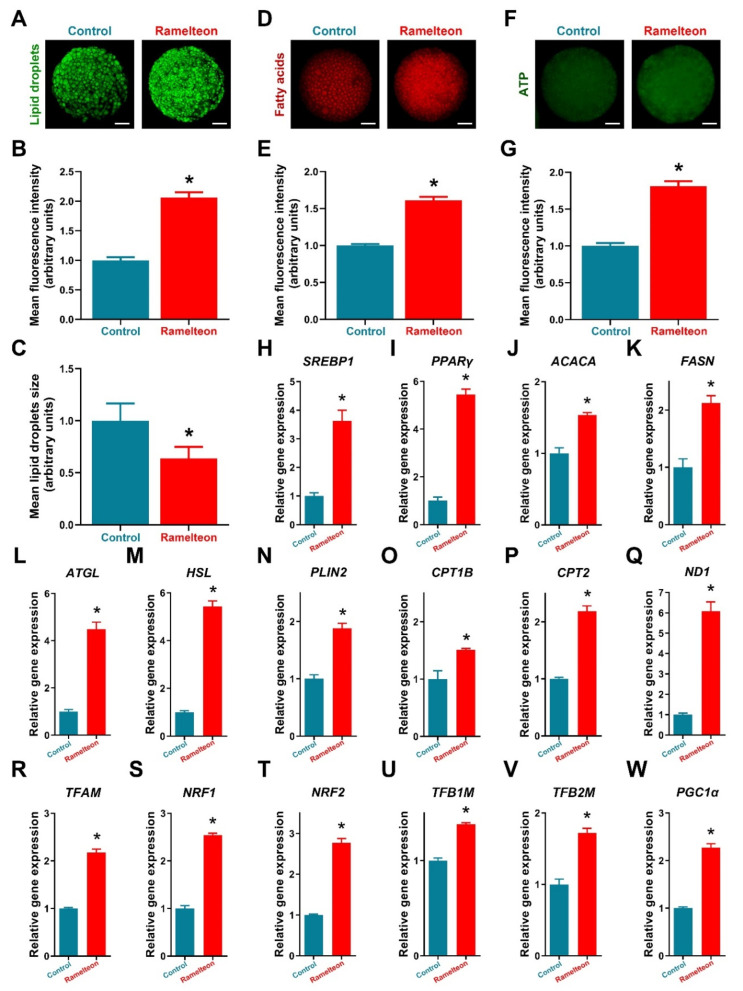
Detection of lipid metabolism−related indicators in porcine oocytes. (**A**–**C**) Lipid droplets intensity and size; (**D**,**E**) Fatty acid contents; (**F**,**G**) ATP contents; (**H**–**W**) Lipid metabolic gene expression. Within the same indicator, bars with an asterisk are significantly different (*p* < 0.05). Results are shown as the average ± SEM of at least three repeats of independent experiments. Ramelteon concentration is 10^−11^ M. Scale bars = 30 μM.

Additionally, treatment of 10^−11^ M ramelteon significantly increased fluorescence intensity represented lipid content in oocytes and significantly reduced the size of LDs compared to the control (Figure 4A–C; *p* < 0.05). Meanwhile, the expression of lipogenesis- (*SREBP1*, *PPARγ*, *ACACA* and *FASN*) and lipolysis-related genes (*ATGL*, *HSL* and *PLIN2*) were significantly improved by 10^−11^ M ramelteon supplementation (Figure 4H–N; *p* < 0.05). In addition, the levels of FAs and ATP were significantly increased in 10^−11^ M ramelteon group compared to the control (Figure 4D–G; *p* < 0.05). Simultaneously, the β-oxidation- (*CPT1B* and *CPT2*) and mitochondrial biogenesis-related gene expression (*ND1*, *TFAM*, *NRF1/2*, *TFB1M/2M* and *PGC1α*) were significantly increased by 10^−11^ M ramelteon supplementation in oocytes (Figure 4O–W; *p* < 0.05).

### 3.5. Ramelteon Improved Oocyte Cytoplasmic Maturation

**Figure 5:**
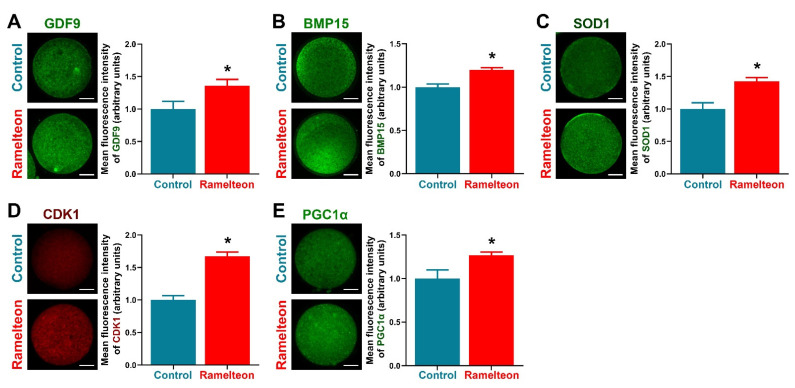
The protein levels were analyzed by immunocytochemistry in porcine oocytes. Expression of (**A**) GDF9; (**B**) BMP15; (**C**) SOD1; (**D**) CDK1; (**E**) PGC1α in oocytes, respectively. Within the same protein, bars with an asterisk are significantly different (*p* < 0.05). Results are shown as the average ± SEM of at least three repeats of independent experiments. Ramelteon concentration is 10^−11^ M. Scale bars = 40 μM.

To determine the effects of ramelteon on oocyte cytoplasmic maturation, several indicators related to cytoplasmic maturation were evaluated. We found that levels of GDF9, BMP15, and SOD1 were significantly improved by 10^−11^ M ramelteon supplementation (Figure 5A–C; *p* < 0.05). Moreover, levels of the maturation-promoting factor (CDK1) and mitochondrial biogenesis protein (PGC1α) were significantly increased in 10^−11^ M ramelteon group compared to the control (Figure 5D,E; *p* < 0.05).

## 4. Discussion

Ramelteon is a selective agonist for MT1 and MT2 melatonin receptors, and has been approved by the Food and Drug Administration (FDA) for the treatment of insomnia in humans [20,21]. Compared with melatonin, it has a greater affinity and a longer half-life at these receptors [22], and it has anti-oxidative and anti-inflammatory effects [23]. Although ramelteon has been associated with an effect on reproductive hormones, its functions and underlying mechanisms on female reproduction are still unclear. In this study, we found that supplementation of an IVM medium with 10^−11^ M ramelteon significantly improved cumulus expansion, nuclear and cytoplasmic maturation of oocytes, and blastocyst formation rate after PA, in comparison to the control. Simultaneously, the expression of cumulus expansion, oocyte and embryo development-related genes was notably increased, and oxidative stress was significantly decreased in 10^−11^ M ramelteon group compared to the control. In addition, according to our results, treatment of 10^−11^ M ramelteon could maintain lipid homeostasis by balancing the lipogenesis and lipolysis in porcine oocytes.

Cumulus expansion is considered to influence a variety of fundamental developmental changes during oocyte maturation [24]. The more intact cumulus cell expansion is critical for protecting oocytes against oxidative stress [25]. Moreover, the bi-directional communications between an oocyte and cumulus cells are indispensable for the oocyte for the acquisition of maturation and early embryonic developmental competence [26]. Therefore, denuded oocytes without cumulus cells exhibit lower oocyte maturation and subsequent embryo development rate [27]. It is well-known that HAS2 is one of enzymes required for hyaluronic synthesis, and PTX-3 and TNFAIP6 mediate the organization of linear molecular of hyaluronan, which has well correlated with the cumulus expansion process [28,29,30]. Moreover, the PTGS1/2 and GLI1 play a crucial role in the acquisition of oocyte competence in cumulus cells [31,32,33,34]. In the present study, supplementation of 10^−11^ and 10^−9^ M ramelteon could promote the CEI significantly, and it appeared to be related to positive regulation of ramelteon on cumulus expansion-related genes including PTX3, PTGS1/2, TNFAIP6 and HAS2. Although the *FGFR2* expression showed no significant difference among groups, the expression of *GLI1* and *BCL2* were upregulated, and anti-apoptosis (*BAX*) was downregulated in 10^−11^ and 10^−9^ M ramelteon groups. These results were in line with exogenous melatonin supplementation [34]. In a previous study, we demonstrated that melatonin improved cumulus cells expansion by increasing cumulus expansion-related genes and sonic hedgehog signaling in pigs [34]. In bovine, supplementation of melatonin during IVM could reduce nuclear fragmentation and apoptosis in cumulus cells, thereby showing cytoprotective effects on COCs from oxidative stress [35]. Taken together, based on the results, we speculated that ramelteon might be critical to the acquisition of maturation and early embryonic development competence.

To demonstrate the hypothesis, we elucidated the effects of ramelteon on porcine oocyte maturation and subsequent embryo development. We found that the oocyte maturation rates were significantly improved by 10^−11^, 10^−9^ and 10^−7^ M ramelteon treatments compared to the control. The relative level of intracellular GSH was significantly increased and the level of intracellular ROS was significantly decreased in 10^−11^, 10^−9^ and 10^−7^ M ramelteon groups. Additionally, the apoptotic genes *BAX* and *BCL2* were, respectively, down- and up-regulated by ramelteon treatment, and the SOD1 protein in oocytes was increased in 10^−11^ M ramelteon group. A previous study suggested that SOD1 deficiency increased superoxide generation, thereby exhibiting abnormal embryo development in murine oocytes [36,37]. Therefore, our results were consistent with most experiments on antioxidant supplementation, which indicated that antioxidants provided advantageous microenvironment to improve oocyte maturation by protecting oocytes against ROS in pigs [19,33,38,39]. Indeed, supplementation of melatonin during IVM could improve oocyte maturation and their subsequent embryo development by reducing oxidative stress and DNA damage in various species [40,41,42,43]. Furthermore, GDF9 and BMP15 are oocyte-secreted factors with a leading role in the control of ovarian function, modulating both the cell fate of the cumulus expansion and oocyte developmental competence [44,45]. The large number of mutations in the *GDF9* and *BMP15* genes have been identified in women with premature ovarian failure and in mothers of dizygotic twin [46]. It is well known that Maturation-promoting factor (MPF) is a key regulator of cell cycles via a complex of CDK1 and CYCLIN B, the activity involves meiotic resumption and oocyte maturation [47]. Our results showed that expression of *GDF9*, *BMP15*, *CYCLIN B1*, *CDK1* and *POU5F1* was up-regulated in 10^−11^ and 10^−9^ M ramelteon supplementations, and the protein levels of GDF9, BMP15 and CDK1 were increased in 10^−11^ M ramelteon group. Moreover, the blastocyst formation rate and total blastocyst cell numbers were significantly improved in 10^−11^ M ramelteon supplementation, but not cleavage rate. Additionally, treatment of 10^−11^ M ramelteon significantly improved hatching/-ed blastocyst rate by up-regulating embryo development-, blastocyst hatching-related gene expression and down-regulating the expression of apoptosis-related genes. Our works provided direct evidence to support the hypothesis that ramelteon do indeed have the capacity to impair oxidative stress and that this beneficial effect might be able to improve oocyte nuclear and cytoplasmic maturation, and their subsequent embryonic development.

Due to oocyte intracellular lipids are providing indispensable energy source to support oocyte maturation [48], it is being increasingly recognized that process of lipogenesis and lipolysis are important for oocyte and their embryo developmental competence [13]. However, excessive formation of lipid droplets during in vitro production of embryos is one of the most well-recognized metabolic abnormalities resulting in energy imbalance, which is a consequence of the increase ROS accumulation during IVM/IVC process [49]. Therefore, the maintenance of lipid content is essential for oocyte maturation and embryo development [11,13]. Our previous study indicated that melatonin promoted lipid metabolism and thereby provided an essential energy source for oocyte maturation and embryo developmental competence [13]. In the present study, 10^−11^ M ramelteon could increase the lipid content by up-regulating lipogenesis-related genes (*SREBP1*, *PPARγ*, *ACACA* and *FASN*) in oocyte. Simultaneously, the size of LDs was reduced by up-regulating lipolysis-related genes (*ATGL*, *HSL* and *PLIN2*) with 10^−11^ M ramelteon supplementation, thereby releasing FAs and undergoing the β-oxidation pathway improving ATP production in oocytes. Interestingly, the expression of mtDNA copy number (*ND1*) and mitochondrial biogenesis-related genes (*TFAM*, *NRF1/2*, *TFB1M/2M* and *PGC1α*) were markedly increased by 10^−11^ M ramelteon treatment. As the previous study, we investigated that melatonin regulated lipid homeostasis in porcine COCs via MT2 [12], whereas, porcine oocytes are only expressed the receptor MT2 [34,50]. Taken together, our results demonstrated that ramelteon reduces oxidative stress by maintenance of lipid homeostasis in porcine oocytes, which might be regulated via MT2.

## 5. Conclusions

Supplementation of 10^−11^ M ramelteon reduces oxidative stress by maintenance of lipid homeostasis during IVM, thereby improving the porcine oocyte maturation and subsequent embryo development.

## Data Availability

All of the data is contained within the article and the Supplementary Materials.

## Supplementary Materials

The following supporting information can be downloaded at: https://www.mdpi.com/article/10.3390/antiox11091640/s1, Table S1: Primer sequences used for real-time PCR.
