# Supplementary material for: Ramelteon Reduces Oxidative Stress by Maintenance of Lipid Homeostasis in Porcine Oocytes
Source: Antioxidants (Basel). 2022 Aug 24;11(9):1640. doi: 10.3390/antiox11091640 (PMC9495855; doi:10.3390/antiox11091640)
Supplement: Supplementary file 1 [file antioxidants-11-01640-s001.zip › antioxidants-1799416-supplementary.pdf]

Table S1. Primer sequences used for real-time PCR

Genes	Primer sequences (5’- 3’)	Product size (bp)	Accession No.
GAPDH	F: GTCGGTTGTGGATCTGACCT<br>R: TTGACGAAGTGGTCGTTGAG	207	NM_001206359
RN18s	F: TCCAATGGATCCTCGCGGAA<br>R: GGCTACCACATCCAAGGAAG	149	NR_046261.1
PTX3	F: GGCCAGGGATGAATTTTAC<br>R: GCTATCCTCTCCAACAAGTGA	185	NM_001244783
PTGS1	F: CAACACGGCACACGACTACA<br>R: CTGCTTCTTCCCTTTGGTCC	121	XM_001926129
PTGS2	F: ACAGGGCCATGGGGTGGACT<br>R: CCACGGCAAAGCGGAGGTGT	194	NM_214321
TNFAIP6	F: AGAAGCGAAAGATGGGATGCT<br>R: CATTTGGGAAGCCTGGAGATT	106	NM_001159607
HAS2	F: AGTTTATGGGCAGCCAATGTAGTT<br>R: GCACTTGGACCGAGCTGTGT	101	AB050389
FGFR2	F: ATTCTGGTGCCGGATGAAGAC<br>R: GGTGTTGGAGTTCATGGAGG	121	NM_001099924
GLI1	F: AGAGGGACAGCTCTGAACAC<br>R: GCTACGTCTCTTCCTCCTGA	199	NM_001256593
BAX	F: TGCCTCAGGATGCATCTACC<br>R: AAGTAGAAAAGCGCGACCAC	199	XM_003127290
BCL2	F: AGGGCATTCAGTGACCTGAC<br>R: CGATCCGACTCACCAATACC	193	NM_214285
GDF9	F: CAGTCAGCTGAAGTGGGACA<br>R: TGGATGATGTTCTGCACCAT	135	AY626786
BMP15	F: CCTCCATCCTTTCCAAGTCA<br>R: GTGTAGTACCCGAGGGCAGA	112	NM_001005155
CYCLIN B1	F: CAACTGGTTGGTGTCACTGC<br>R: TTCCATCTGCCTGATTTGGT	126	L48205
CDK1	F: GGGCACTCCCAATAATGAAGT<br>R: GTTCTTGATACAACGTGTGGGAA	260	AB045783
POU5F1	F: TTTGGGAAGGTGTTCAGCCAAACG<br>R: TCGGTTCTCGATACTTGTCCGCTT	198	NM_001113060
NANOG	F: GGTTTATGGGCCTGAAGAAA<br>R: GATCCATGGAGGAAGGAAGA	98	NM_001129971
SOX2	F: ATGCACAACTCGGAGATCAG<br>R: TATAATCCGGGTGCTCCTTC	130	NM_001123197
CDX2	F: TGTGCGAGTGGATGCGGAAG<br>R: CCGAATGGTGATGTAGCGAC	149	NM_001278769
PCNA	F: CCTGTGCAAAAGATGGAGTG<br>R: GGAGAGAGTGGAGTGGCTTTT	187	XM_003359883

GLUT1	F: GCTTCCAGTATGTGGAGCAA<br>R: AAGCAATCTCATCGAAGGTC	132	XM_013977359
DNMT1	F: TCGAACCAAAACGGCAGTAC<br>R: CGGTCAGTTTGTGTTGGACA	215	NM_001032355
DNMT3A	F: CTGAGAAGCCCAAGGTCAAG<br>R: GTACTGATACGCGCACTCCA	200	NM_001097437
DNMT3B	F: AGTGTGTGAGGAGTCCATTG<br>R: GCTTCCGCCAATCACCAGT	133	XM_013985277
NR5A2	F: GGTACCACTATGGGCTCCTCAC<br>R: TCGGCCCTTACCGCTTCT	193	NM_001267893
IL-1β	F: GCCAATGGTTTTCTCTGTGATGCC<br>R: CTCATGCAGAACACCACTTCTCTC	158	NM_001302388
TFAM	F: TCCGTTCAGTTTTGCGTATG<br>R: TTGTACACCTGCCAGTCTGC	240	NM_001130211
PRDX2	F: TGTCCTTCGCCAGATCACT<br>R: TCCACGTTGGGCTTGATT	156	NM_001244474
CASPASE3	F: CGTGCTTCTAAGCCATGGTG<br>R: GTCCCACTGTCCGTCTCAAT	186	NM_214131
SREBP1	F: ACCCGCTTCTTCCTGAGTA<br>R: ACGGAACAACTGAGTCACCT	207	NM_214157
PPARγ	F: AGAGCTGATCCAATGGTTGC<br>R: GAGTTGGAAGGCTCTTCGTG	146	NM_214379
ACACA	F: AACAAGGACCTGGTGGAGTG<br>R: GTCATGTGCACGATGGAATC	170	NM_001114269
FASN	F: AACTTCCGAGACGTCATGCT<br>R: GTGCTGAAGCAGCAGAACAG	180	NM_001099930
ATGL	F: CGAACTCAAGAGCACCATCA<br>R: TTGCACATCTCTCGAAGCAC	189	NM_001098605
HSL	F: TGTCTTTGCGGGTATTCG<br>R: TTGTGCGGAAGAAGATGC	209	NM_214315
PLIN2	F: TGTGAGATGGCAGAGAAGGG<br>R: CACAGCCCCTTTAGCATTGG	198	NM_214200
CPT1B	F: ATCAAGCCTGTGATGGCTCT<br>R: GAGCCACACCTTGAAGAAGC	168	NM_001007191
CPT2	F: AGTTCCAGAGAGGAGGCAAAG<br>R: GAGCATCTCTTGGTGAAGACG	199	NM_001246243
ND1	F: TCCTACTGGCCGTAGCATTCCT<br>R: TTGAGGATGTGGCTGGTCGTAG	165	808501
NRF1	F: ACCATCCAGACAACGCAA<br>R: ACTCCAGTAAGTGCTCCGAC	230	XM005657993
NRF2	F: GCCCAGTCTTCATTGCTCCT<br>R: AGCTCCTCCCAAACTTGCTC	115	XM_013984303
TFB1M	F: CGAGGGCTTGGAATGTTA<br>R: CGTGTGCCTGAGTTCTTCT	204	NM_001128475

TFB2M	F: GCAAGGAGGAAGGATGTT<br>R: CAAGTAATGCTCGTGTCAGG	243	XM_001927064
PGC1α	F: TTCCGTATCACCACCCAAAT<br>R: ATCTACTGCCTGGGGACCTT	137	NW_213963

F, Forward primer; R, Reverse primer.
